# The Effect of Physiological Incubation on the Properties of Elastic Magnetic Composites for Soft Biomedical Sensors

**DOI:** 10.3390/s21217122

**Published:** 2021-10-27

**Authors:** Joanna Mystkowska, Anna Powojska, Dawid Łysik, Joanna Niewęgłowska, Gilbert Santiago Cañón Bermúdez, Arkadiusz Mystkowski, Denys Makarov

**Affiliations:** 1 Institute of Biomedical Engineering, Bialystok University of Technology, Wiejska 45C, 15-351 Bialystok, Poland; a.powojska@doktoranci.pb.edu.pl (A.P.); d.lysik@pb.edu.pl (D.Ł.); nieweglowska.asia@gmail.com (J.N.); 2 Helmholtz-Zentrum Dresden-Rossendorf e.V., Institute of Ion Beam Physics and Materials Research, Bautzner Landstrasse 400, 01328 Dresden, Germany; g.canon-bermudez@hzdr.de (G.S.C.B.); d.makarov@hzdr.de (D.M.); 3 Department of Automatic Control and Robotics, Faculty of Electrical Engineering, Bialystok University of Technology, Wiejska 45D, 15-351 Bialystok, Poland; a.mystkowski@pb.edu.pl

**Keywords:** soft robot, biomedical sensor, magnetic composite, elastic modulus, DSC, TGA

## Abstract

Magnetic micro- and nanoparticles (MPs)-based composite materials are widely used in various applications in electronics, biotechnology, and medicine. This group of silicone composites have advantageous magnetic and mechanical properties as well as sufficient flexibility and biocompatibility. These composites can be applied in medicine for biological sensing, drug delivery, tissue engineering, and as remote-controlled microrobots operating in vivo. In this work, the properties of polydimethylsiloxane (PDMS)-based composites with different percentages (30 wt.%, 50 wt.%, 70 wt.%) of NdFeB microparticles as a filler were characterized. The novelty of the work was to determine the influence of the percentage of MP content and physiological conditioning on the properties of the PDMS-MP composites after in vitro incubation. An important essence of the work was a comprehensive study of the properties of materials important from the point of view of medical applications. Materials were tested before and after conditioning in 0.9 wt.% NaCl solution at a temperature of 37 °C. Several studies were carried out, including thermal, physicochemical, and rheological tests. The results show that with an increase of the incubation time, most of the measured thermal and physicochemical parameters decreased. The presence of the magnetic filler, especially at a concentration of 70 wt.%, has a positive effect on thermal stability and physicochemical and rheological properties. The performed tests provided important results, which can lead to further research for a broader application of magnetic composites in the biomedical field.

## 1. Introduction

In recent years, elastomeric composites with magnetic functionalities have attracted much attention of the research community. Fabrication of smart materials and miniaturization are key for developing modern electronics, robotics, health care systems, and biomedical engineering [1,2]. There is a great potential in polymer-based materials reinforced with magnetic particles, as their properties can be readily tuned by the strength and direction of external magnetic fields, which act at a distance [3,4,5,6,7,8]. Remotely controlled structures are very promising and can have an impact on the evolution of lab-on-a-chip sensors, targeted drug delivery and medical soft robotics [9,10,11]. One of the polymers used as a matrix for soft robots is polydimethylsiloxane (PDMS), which is a silicone in the form of fluid widely used for wearable electronics, microfluidic chips, and for biomedical applications. Among its outstanding advantages are transparency, elasticity, biocompatibility, and stable chemical properties [12]. There are many studies considering this material in applications connected with treatment, medical instrumentation, prosthetics, and more. Qi et al. described an application of PDMS for stretchable electronics which can be useful not only for industrial use, but also for manufacturing in healthcare [13,14]. Chen et al. proposed a flexible pressure sensor made out of PDMS and transparent electrodes [15]. PDMS was also used by Ozbolat et al. for the 3D printing of human organ models, as human nose, ear, or even internal structures [16]. A developing trend in biomedical engineering is the use of PDMS for the production of microrobots for microsurgery and the fabrication of structures and drug delivery in hard-to-reach areas in the human or animal body [17,18,19,20,21].

There are various substances used as reinforcement for soft, elastic composites, such as magnetic particles, which provide material control by means of a magnetic field. Among the currently known filler materials, iron oxide micro- and nanoparticles (Fe_3_O_4_, CoFe_2_O_4_) and NdFeB are widely used to reinforce composites and grant magnetic properties [22,23,24,25]. NdFeB particles demonstrate hard ferromagnetic properties with density in a range 7.5–7.6 g/cm^3^ [26]. The form (particles, tapes, fibers), shape (spherical, irregular), and application of used reinforcement determine the mechanical and physicochemical properties of magnetic materials [27]. Magnetic particles (MPs) of certain oxides such as ZnFe_2_O_4_ play a role in the diagnostics and treatment of cancer and bacterial infections [28]. Furthermore, magnetic resonance imaging (MRI) and computer tomography (CT) can be enhanced using magnetic particles, which accumulate in a pathologically changed tissue and facilitate the diagnostic of a problem immediately [29,30,31]. The improvement of existing applications as well as advanced solutions in the area of tissue engineering and targeting therapies are the future perspective for MPs [23,32].

In numerous fields, such as microrobots, sensors, and drug delivery systems, there is a need for hydrophobic materials with a smooth surface, and stable mechanical and physicochemical properties. Low roughness and high contact angle (above 90°) can help avoid cell proliferation or even act as an antibacterial surface [14,33]. However, there are also other applications, mainly in the tissue engineering field, where fine roughness and hydrophilicity are demanded in terms of tissue and cell culture for proper osteointegration [34]. Therefore, determining material parameters such as density, wettability, thermal stability, and elastic modulus is essential before combining these artificial composites with natural materials [35], particularly during in vitro tests. If there is any influence of the contact medium on the material properties, it should be carefully analyzed if the magnetic composite may be considered for medical applications [36,37]. Investigating thermal properties of the composites, such as the glass transition temperature *T_g_* and melting point *T_m_* after incubation, provides information about ongoing decomposition processes, the nature of material and the stability of its mechanical properties [38,39]. Above *T_g_* there is a significant change in viscosity and at *T_m_* a transition between the solid and liquid phase occurs in the material. Thermal analyses are, in addition to mechanical, rheological, and biological tests, some of the most important analyses to be carried out. Understanding the thermal characteristics of the composite, especially for biomedical applications, is the key to preparing new material composition and fabrication. Thermal analysis is helpful in determining maximum temperature in which composite maintains its properties, so the sterilization or surface modification can be performed without affecting the material properties [40,41]. Analysis of the above results may be a starting point for further experiments, including modifications of the chemical composition, surface modifications and biological treatment [42].

The stability of the mechanical properties of PDMS-MP composites is very important for medical applications. The mechanical stability of magnetic materials is mainly determined by (1) external forces caused by tissue pressure and cell response to the stiffness of PDMS-MPs substrates [43], and (2) internal forces caused by MP movement in material in contact with an electromagnetic field [44]. PDMS is a viscoelastic polymer, which means that, during deformations, part of the mechanical energy is stored in the material and part of the energy is dissipated [45]. In PDMS-MP composites, the mechanical characteristics depend not only on the polymer matrix, but also on the MP content, and in the in vivo applications, they may change dynamically due to the influence of the tissue environment. The PDMS-NdFeB composites were used for further possibility of magnetization, since the NdFeB is the most used powder of the strongest type of permanent magnet available commercially and has the highest magnetic energy density. Therefore, the magnetized PDMS-NdFeB composites can be used as the magnetic actuators in applications, where the actuator movements are controlled using the external active magnetic field.

There is not enough information in the literature, describing thermal, physicochemical, and rheological properties of NdFeB composites to in vivo applications. Due to our best knowledge and overview of the literature data, there are no publications where the above-mentioned properties have been comprehensively tested for PDMS-MP composites intended for medical applications. The results of the tests presented in this paper are the first step to further research of this group of composites in the biomedical field. The primary focus of this work was to investigate the effect of the MP content on the properties of the PDMS-MP-based composites. The second goal was to explore how incubating the composites in physiological fluid influences their thermal, physicochemical, and mechanical properties. Properties such as wettability, water absorption, change of thermal properties, or stiffness are very important because they change the mechanical parameters, which is important from the point of view of the durability of materials and their performance properties in the human body.

## 2. Materials and Methods

### 2.1. PDMS-MPs Preparation

Elastomeric silicone composites reinforced with metallic micropowder were prepared. The organic matrix was a two-component Sylgard 184 PDMS silicone (Dow Corning, USA) and a metal alloy with magnetic properties (MQFP-14-12-20000-088, Magnequench, Singapore, Singapore) was used as a filler. Magnetic microparticles (MPs) size distribution is given by the manufacturer as d_50_ = 25 microns. Composition of metal powder is shown in Table 1.

**Figure 1:**
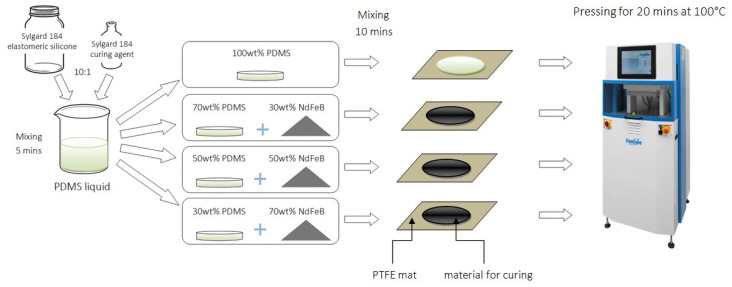
Scheme of the fabrication process of PDMS-MPs composites.

Schematics of the preparation of the composite are shown in Figure 1. First, the liquid silicone elastomer was mixed with a curing agent in a weight ratio 10:1, according to information provided by the supplier. Next, the prepared matrix was divided into four parts. Three of them were mixed with magnetic particles in percentages of 30 wt.%, 50 wt.%, 70 wt.%, the fourth one was a control material (clear PDMS). Composites were prepared by hand mixing in a plastic container, and then poured on a PTFE mat. Materials in a form of liquid were put into a vacuum pump to perform gas removal. Thanks to this, the resulting materials were devoid of gas-filled spaces. Clear PDMS and composites were placed respectively in a simple mold in a LabEcon 300 hydraulic press (Fontijne, Vlaardingen, The Netherlands) to fasten the curing process and provide a thickness of 1 mm for all materials. The curing process was performed in the temperature of 100 °C for 20 min for each material. The resulting material was in a form of thin sheets.

As incubation medium, a solution of 0.9 wt.% Sodium Chloride (NaCl, Sigma Aldrich, St. Louis, MO, USA) in ultrapure Milli-Q water (Merck Milipore, Darmstadt, Germany) was used. Materials were cut in small pieces, weighted and placed in sealed plastic containers. Each container for incubation included a material sample and medium in a weight ratio 1:10. Conditioning studies were performed in an incubator with an internal temperature range of *T* = 37 ± 0.5 °C. The materials were incubated for time intervals of *t* = 1, 2, 4, 8 and 12 weeks. Each sample, taken out of the container, was dried at room temperature for 24 h (*T* = 22 °C, humidity 50%) before performing the experiments. Designations of samples adopted in the paper are shown in Table 2.

### 2.2. Thermal Properties

DSC tests were carried out using DSC Discovery apparatus (TA Instruments, New Castle, DE, USA). The experiments were conducted according to the guidelines of the ISO 11357–1:2016 standard [46] in three parts (Heat–Cool–Heat) in a temperature range from −90 to 250 °C in a nitrogen atmosphere. The heating and cooling rate was of 10 °C/min. The results presented and discussed in the paper were taken from the second heating curve. Three samples of each material were analyzed.

TG studies were carried out using a Q500 thermogravimetric analyzer (TA Instruments, USA). The experiment was performed in accordance with the guidelines of the ISO 11358–1:2014 standard [47]. The measurements were conducted in a nitrogen atmosphere and a temperature range from 30 to 950 °C. The heating rate (*k*) was 10 °C/min for all of samples, and additionally 5 °C/min and 20 °C/min for samples 70-0 and 70-12 to calculate the activation energy of thermal decomposition. The value of activation energy was calculated using the Kissinger method [48] expressed by Equation (1):

(1) $$\ln\left(\frac{k}{T_{max}^{2}}\right)=-\frac{E_{a}}{R}\times \frac{1}{T_{max}}+const.$$

where *E_a_* is the activation energy, *R* is the gas constant equal to 8.31 J/mol∙K and the maximum temperature *T_max_* increases proportional to the heating rate *k*. The Kissinger method assumes the presentation of the obtained data as ln(*k/T_max_*^2^)~1*/T_max_*. The directional coefficient of the resulted line graph is equal to the *E_a_/R* [39].

Both DSC and TGA test were performed using of 5 mg of material in form of small pieces in a solid state. PDMS and its composites are very elastic and easy-to-cut materials. The preparation of samples of remaining weight was performed using a sharp scalpel. Among the obtained results, special attention was paid to a 1 wt.% and 5 wt.% loss of the samples, as well as the lowest weight at the end of the decomposition process. In addition, we recorded the temperature at which the highest rate of weight loss was attained. Three samples of each material were analyzed.

### 2.3. Physicochemical Properties

A Contact Angle Goniometer (Ossila, Sheffield, UK) was used to determine the wettability of the surface. Wettability was described by the contact angle (*Ѳ*) for clear PDMS and PDMS-MPs composites. The contact angle was measured between the surface of the examined material and a 5 µL droplet of ultrapure water on the surface. The acquired images were analyzed with the Ossila Contact Angle software, which uses a tangent method for measurements [35]. For each sample, contact angle was measured using three droplets of the same volume, located in different places on the examined material. Obtained results were averaged for three samples.

To measure the water absorption (*W*), each sample was weighted before immersing in medium and after a set incubation time followed by 24 h of drying at room temperature (*T* = 22 °C, 50% humidity). To measure the weight of the samples, a balance with a sensitivity of 0.01 mg was used (Mettler Toledo, Columbus, OH, USA). The water absorption is a percentage gain weight, considering the initial dry weight of the sample (*w_d_*) and weight of the sample after incubation (*w_w_*). It was calculated from the following Equation (2).

(2) $$W\left(\%\right)=\frac{w_{w}-w_{d}}{w_{d}}\times 100\%$$

The density (*d*) of the examined materials was determined using the hydrostatic method, based on the weight of the samples in two environments of known density. A balance (Mettler Toledo, Columbus, OH, USA) with dedicated equipment for density measurements was used. Each sample was placed first on a plate in air, then in the container filled with ultrapure water. The weight of the sample in the air and in water was recorded by the balance. The device automatically calculated the density of the analyzed sample using Archimedes’ principle. The measurements were performed five times for each sample.

The surfaces of the examined materials were observed using a Confocal Laser Scanning Microscopy (CLSM) technique. The microscope used in the experiment was LEXT OLS 4000 (Olympus, Tokyo, Japan). This non-destructive method with real-time imaging uses laser and white light to give a three-dimensional representation of the measured surface. Before performing the observations, each sample was rinsed in ultrapure water to remove possible surface contamination. The surface roughness was calculated by the software after imaging. For each sample five measurements were taken.

### 2.4. Rheological Properties

**Figure 2:**
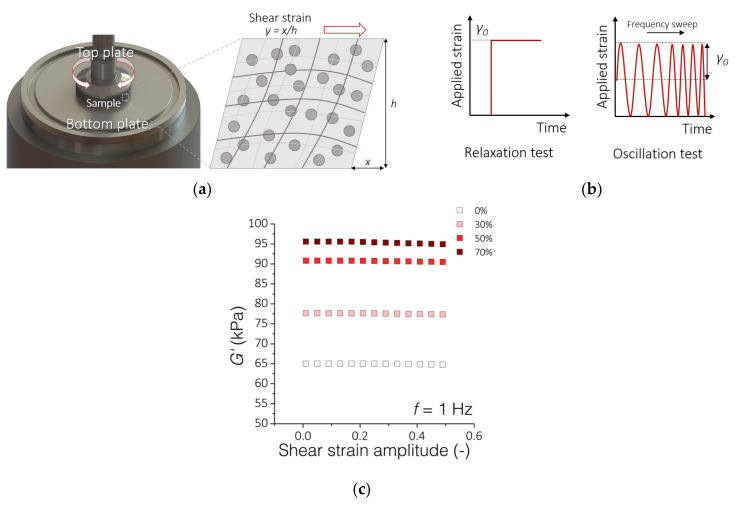
Rheological experimental setup. (**a**) Measuring system for testing the rheological properties of PDMS-MPs composites; (**b**) The scheme of material deformation overtime during the performed rheological tests; (**c**) Results of oscillatory tests with variable amplitude of shear deformations and a constant frequency *f* = 1 Hz. The graph shows the range of linear viscoelasticity up to about 20% of the deformation amplitude.

Rheological analyses were carried out on a HAAKE Rheostress 6000 rheometer equipped with a Peltier temperature control system (Thermo Fisher Scientific, Waltham, MA, USA) (Figure 2a). For the tests, we used disk-shaped PDMS-MPs samples with a diameter of 20 mm and a thickness of 1 mm. All measurements were performed at 37 °C in a plate–plate configuration (20 mm diameter of the top plate). First, the composite sample was placed on the non-slip bottom plate, then the top rheometer plate was lowered until an initial force of 0.1 N was obtained. The distance between the top and bottom plates was the measurement gap h ~ 1 mm. The rheological analyzes are based on two tests (Figure 2b). The first one consisted of forcing a constant 10% shear strain *γ* in the material and measuring the stress response for 600 s. The stress-to-applied strain ratio was defined as the relaxation modulus *G*. The second test consisted of deforming the sample in an oscillating manner with a constant amplitude of *γ_0_* = 1% and a variable frequency *f* from 0.1 to 10 Hz. Based on the obtained stress waveform, the dependence of the storage modulus *G*′ as a function of frequency was determined. To better describe the results, we adjusted the parameters (*G_0_*, *G_1_*, *v_1_*) of the standard linear solid model to the obtained data. The range of deformations used in these tests is within the range of the linear viscoelasticity of these materials as shown in Figure 2c.

## 3. Results and Discussion

### 3.1. Thermal Analysis

The thermal properties of materials are important not only at the stage of their preparation, but also during application. It should be taken into account that a change in thermal properties, which are tested at much higher temperatures than the human body, carries information about a change in other properties, including mechanical or physicochemical. Among the tested parameters is activation energy of material decomposition, the value of which allows estimation of the susceptibility of the material to changes in its operational properties in the tested environment. In this case it was a simulated physiological environment. This information, in turn, shows that some changes are taking place in the material, which may have application implications in the context of estimating the durability of material properties.

**Figure 3:**
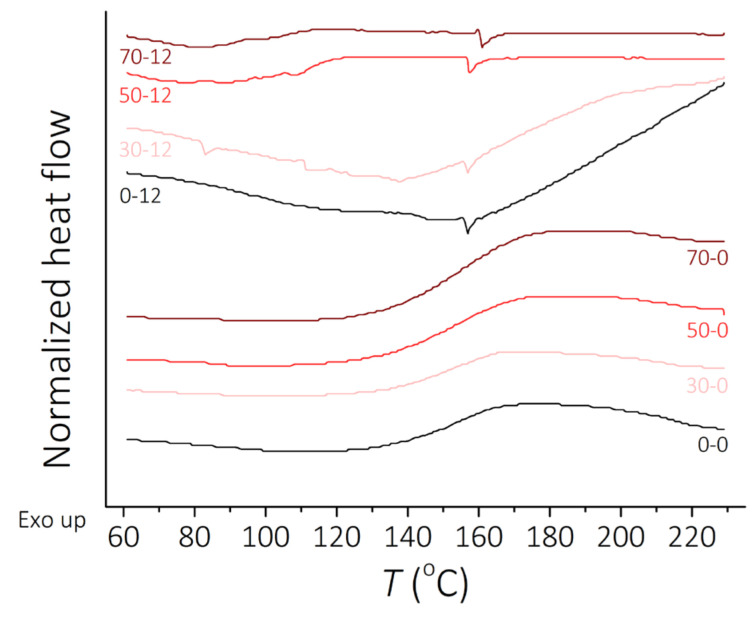
DSC curves, heat flow vs. temperature. Curves for the second heating before incubation and after 12 weeks of incubation.

The results of DSC tests for the clear PDMS polymer and PDMS-MPs composites before and after incubation in a 0.9 wt.% saline solution for 12 weeks are presented in Figure 3. Analysis of the obtained DSC spectra allowed the determination of exothermic peaks observed in the range 140–170 °C for the second heating experiment, probably corresponding to the cyclization reactions of the PDMS [49,50], which were observed mainly for samples before incubation and endothermic peaks around 150–160 °C for materials after 12 weeks of incubation. The typical exothermic peak gives important information for describing the curing phenomenon. It is observed that 12 weeks of incubation affects the PDMS, revealing a different kind of reaction. Exothermic peaks observed for non-incubated samples changes to endothermic peaks for incubated materials. The presence of MPs in composite raises the temperature of characteristic transitions by around 10 °C. DSC analysis allows adjustment of a percentage of the filler in proposed composite material so it can affect thermal properties positively. Unfortunately, there was no chance to observe glass transition temperature (*T_g_* around −120 °C) of PDMS [51] with the type of equipment used in the experiment.

**Figure 4:**
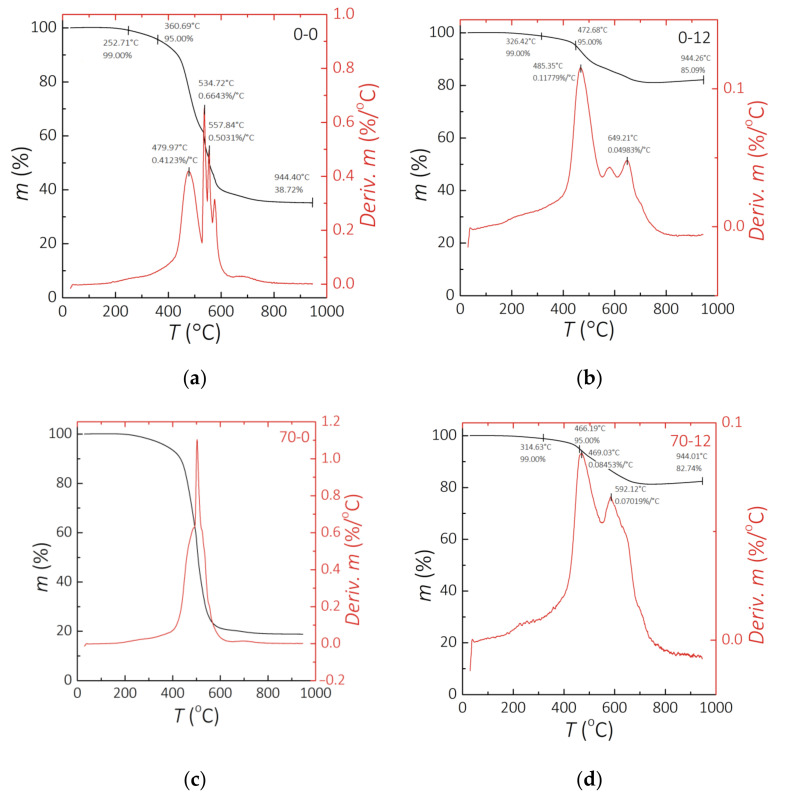
Results of the TG analysis. (**a**) 0-0 sample. (**b**) 0-12 sample. (**c**) 70-0 sample. (**d**) 70-12 sample.

Figure 4 presents the results of the TG analysis. The left and right *y*-axes of the graphs respectively show the residue of material in % and the speed of weight loss as a function of temperature. For most of the performed experiments it was possible to observe two or three peaks of intensive weight loss. The residue, represented by a black line, has its lowest value not at the end, but 200 °C before the end of analysis. It is observed for the PDMS-MPs composites. The increase of weight can be caused by the formation of nitrogen compounds [52], as the experiment was performed in a nitrogen atmosphere, which will be verified in the following experiments. It can be observed that the residue in clear PDMS is much lower than in 70 wt.% MPs composite. Furthermore, the residue for 70 wt.% composites between 70-0 and 70-12 samples changes by less than 3%. The residue for clear PDMS from 0-0 to 0-12 changes by around 20%. This is visible difference which leads to the conclusion that the incubation process affects clear PDMS thermal stability, but those composites maintain their thermal properties. The thermal decomposition process is 10 times more dynamic for clear PDMS than for composites with 70 wt.% of filler. The speed of weight loss in Figure 4a,b represented by a red curve shows three or more peaks for intensive weight loss of the 0-0 and 70-0 samples, respectively. The results of the same value presented in Figure 4c,d consist of one or two peaks of the weight loss of 0-12 and 70-12 samples. The incubation time reduces processes of instantaneous weight loss, represented by a reduced number of peaks. The weight loss process for samples after incubation is less dynamic, but it runs for higher values of speed of weight loss for a wider temperature range. This can cause faster decomposition of the material after incubation, both for a clear PDMS and PDMS-MPs composites.

**Figure 5:**
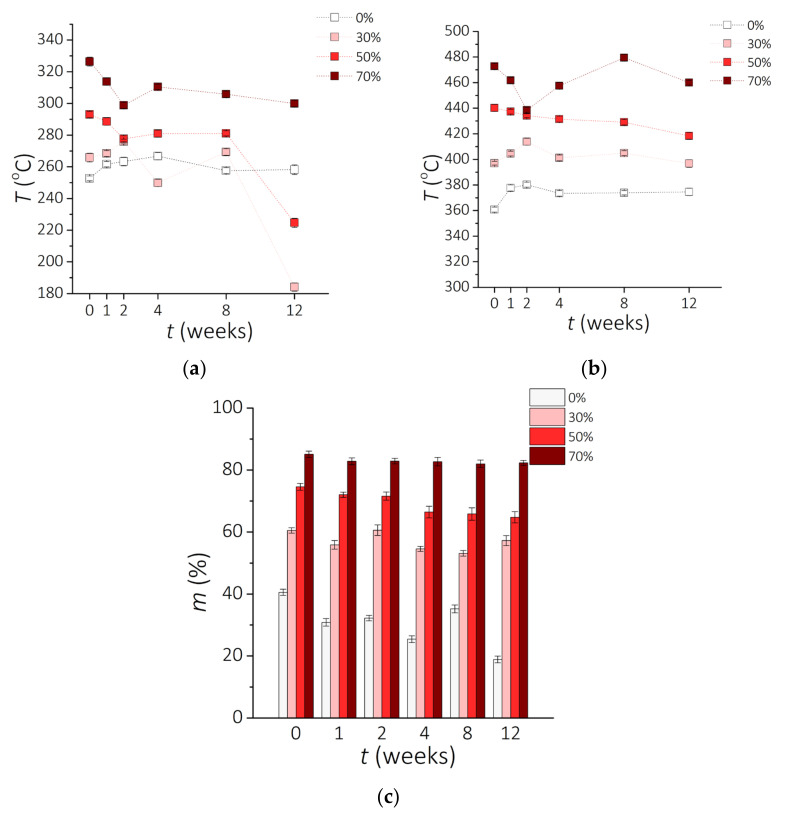
Results of the TG analysis. Temperature *T* for (**a**) 1 wt.% and (**b**) 5 wt.% loss after incubation time *t* during the TG analysis. (**c**) Residue of sample *m* after incubation time *t*.

The TG curves given in Figure 5 present the data derived from the thermogravimetric analysis (TGA) curves for a clear PDMS and PDMS-MPs composites. Figure 5a indicates that the composite with 70 wt.% addition of NdFeB MPs improves the thermal stability of the material, and that PDMS-MPs starts depolymerizing (mass loss of 1 wt.%) around 300 °C and completes at about 600 °C, leaving some residue. Results obtained for 30 wt.% and 50 wt.% composites show that there are no visible changes in their thermal stability up to 8 weeks of incubation. Surprisingly, quite a large decrease of temperature for 1 wt.% loss is observed for samples incubated for 12 weeks with different powder content: 30-12 (decrease of 64.12 °C) and 50-12 (decrease of 82.95 °C) compared to previous weeks (Table 3). The 5 wt.% loss of the initial sample weight (Figure 5b) is obtained at the same range of temperature for a clear PDMS and for composites containing 30 wt.% and 50 wt.% of MPs. The trend for 70 wt.% composite is not linear and varies in time. It is observed that with higher percentage of the magnetic filler, the temperature for thermal decomposition is higher. The thermal decomposition of PDMS takes place in two stages, where according to the literature data [53,54] the liberation of cyclic oligomers occurs. It was graphically discussed in the work of Nair et al. [53] in that the formation of cyclic oligomers from the linear polymer suggests the breaking of the Si-O bond in the polymer.

The residue material after the TG analysis for samples before and after conditioning is shown in Figure 5c. The percentage of weight remaining for a clear PDMS and composites with 30 wt.% of MPs vary over time of incubation. For clear PDMS, the residue without incubation vs. after 12 weeks of incubation decreased from 39.7% for a 0-0 sample to 18.4% for 0-12 sample. Results presented by Nair et al. shows that the PDMS decomposition was completed at about 590 °C, leaving no residue [53]. Sethy et al. presents TG analysis for PDMS, where the decomposition process is similar to the one obtained in our study [55]. Moreover, the authors show that the additives enhance the thermal stability of the PDMS matrix, as observed in this experiment as well. The higher value of the residue of PDMS-MPs composites could be assigned to the presence of inorganic compounds of NdFeB microparticles. Metal is able to sustain even at high temperatures [56].

From TG curves, temperatures of thermal decomposition for 1 wt.% material loss (*T_1wt%_*) and 5 wt.% (*T_5wt%_*) material loss are identified as well as mass residue at 950 °C and temperature of the highest material loss peak (*T_peak_*) and the value of maximum speed of weight loss (*Deriv. m*) were noticed. Data derived from TG experiments are shown in Table 3. The speed of decomposition process is a few times higher for PDMS than for PDMS-MPs composites. For samples after 12 weeks of incubation in 0.9 wt.% saline solution it is observed that maximum speed of decomposition of 0-12 sample is 5 to 10 times higher than for samples 30-12, 50-12 and 70-12. This value is represented on the TG graphs as the highest peak on the right *y*-axis. The presence of magnetic microparticles can slow down the decomposition process. Reduction reaction dynamics during heating for composites vs. clear PDMS is observed.

Table 4 presents the activation energy of the thermal decomposition for 70 wt.% composite, just after the composite synthesis (70-0) and after 12 weeks of incubation (70-12). The results clearly show that after incubation, the activation energy for the composite 70-12 is more than 3 times lower in comparison to the material 0-0 before incubation, which means that less energy is required to initiate thermal decomposition of this material. The observed decrease in the activation energy indicates degradation of the thermal stability. Thanks to the obtained data, it is possible to understand the characteristics of the PDMS-MPs-based composites regarding the thermal decomposition. The lower the *E_a_* value, the less complicated a thermal decomposition process is observed [39,57].

### 3.2. Physicochemical Properties

**Figure 6:**
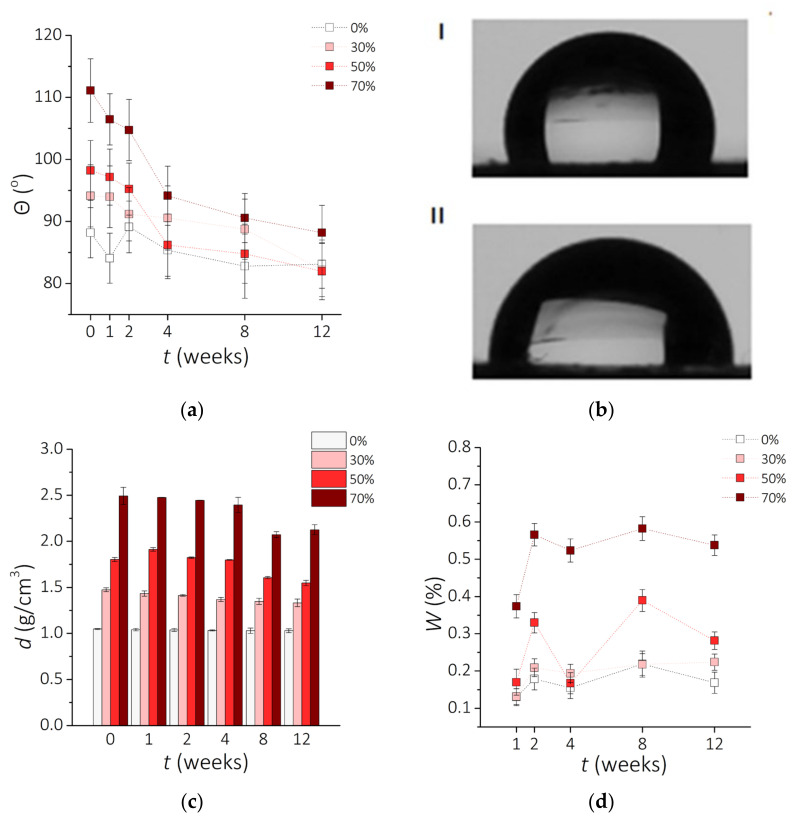
Physicochemical properties of the examined materials vs. incubation time *t*. (**a**) The change of the contact angle *θ* with the incubation time (±SD, *n* = 5). (**b**) Images of water droplets for two composites PDMS—70 wt.% MPs: before incubation 70-0 (I) and after 12 weeks of incubation 70-12 (II). (**c**) Changes of the density *d* of the composite with time of incubation (±SD, *n* = 5). (**d**) Water absorption *W*.

Figure 6a presents the effect of the incubation time and the NdFeB particle content on the contact angle. Analysis of the obtained data shows that clear PDMS is hydrophilic; however, composites based on this silicone are hydrophobic. All analyzed samples tend to be hydrophilic after 12 weeks of incubation and show a decrease in the contact angle as the incubation time is increased. Composites with 70 wt.% NdFeB MPs were the most hydrophobic; the 70-0 sample is characterized by the contact angle *Ѳ* = 111°, which decreased to *Ѳ* = 91° after 12 weeks of incubation (Figure 6b). Other samples followed a similar trend, although the change with time was not as pronounced. Before incubation, samples with higher percentage of NdFeB micropowder displayed higher contact angles. However, after 12 weeks, this correlation was noticeably reduced, and samples 0-12, 30-12, and 50-12 converged towards the contact angle of 85°. It was noticed that hydrophobic surfaces could turn hydrophilic after 4 to 12 weeks with no additional surface modification. It must be considered that for cell adhesion and proliferation more hydrophilic surface is beneficial. For the examined composites surface modifications will be necessary to adapt them to tissue-engineering applications. The obtained results for PDMS-based materials are lower than reported previously (*Ѳ* = 107° or *Ѳ* = 116° for clear PDMS) [58,59,60], which could be explained by the differences in the fabrication method [61].

Results obtained for the sample density during incubation are shown in Figure 6c. The highest density was observed for the 70-0 composite (*d* = 2.493 g/cm^3^) and the lowest for 0-12 clear PDMS (*d* = 1.028 g/cm^3^). The value for the clear PDMS is consistent with the literature data (1.02–1.04 g/cm^3^ [62]). Naturally, it is expected that the presence of metal particles increases the final density of the prepared PDMS-MPs composites. However, the changes in density are more visible for composites with higher MPs concentration. The value for the clear PDMS is more stable with the time of incubation. This could be probably caused by chemical reactions between sample and 0.9 wt.% NaCl solution. Presumably, the presence of contact solution contributed to rinsing out magnetic particles. The solution could go deeper into the material and cause composite cross-linking, especially in the case of high MP concentration. The internal reconstruction processes could reduce the density of material [63]. Incubation leads to a decrease in density of material more for composites with a high percentage of NdFeB particles. This change in density can influence the internal structure. Upon incubation, the density of the composites with 30, 50, and 70 wt.% of NdFeB tended to decrease. However, it was mostly evident for the sample 70-0, which decreased from *d* = 2.494 g/cm^3^ to *d* = 2.126 g/cm^3^ after 12 weeks. Similarly, between samples 50-2 and 50-12 there was a decrease in the density from *d* = 1.804 g/cm^3^ to *d* = 1.548 g/cm^3^. There was only a slight distinction of the density between samples 30-0 and 30-12 as well as between 0-0 and 0-12. According to these results, composites with a higher percentage of NdFeB particles seem to have a more significant decrease in density. This tendency could be attributed to the metallic filler of the composite being dissolved in the salt solution due to electrochemical corrosion [64].

The results of the water absorption (*W)* experiments as a function of the incubation time are presented in Figure 6d. The lowest percentage of the change between 0.1–0.2% was observed for a clear PDMS, which is expected to absorb almost no water at all [35,61,65]. Increasing the concentration of MPs correspondingly raised the weight of the composites, especially for 50 wt.% and above, reaching up to 0.6% weight increase for 70 wt.%. It must be considered that the highest percentage obtained is a very small value (less than 1% of initial weight) and does not affect the weight of the sample at all. However, the water absorption parameter still must be considered.

**Figure 7:**
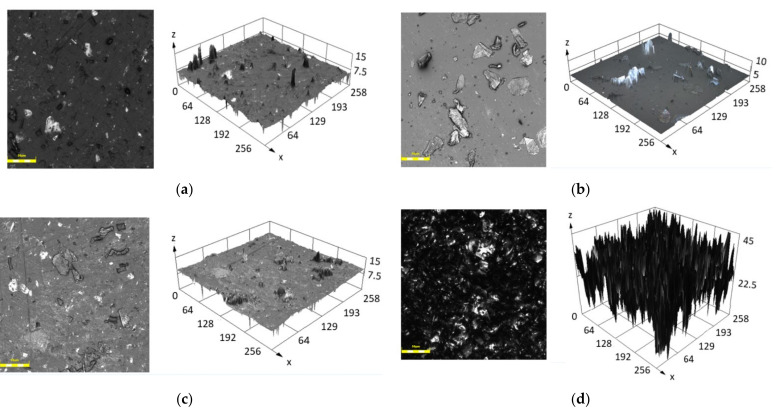
CLSM analysis of the examined surfaces for the samples, 2D and 3D view: (**a**) 50-0, (**b**) 70-0, (**c**) 50-12, (**d**) 70-12. Scale bar ~50µm.

The results of the surface roughness (*Sa*) measured with CLSM are shown in Table 5. The roughness of the surfaces of a clear PDMS is about *Sa* = 0.1 µm, which corresponds to the results available in the literature [61,66]. For all the examined composites, *Sa* value is higher after incubation (Figure 7) and the largest changes are found for samples 70-0 and 70-12 (830 µm) as well as for 0-0 and 0-12 (650 µm). This increase in the surface roughness could be attributed to the interaction of the saline medium with polymer matrix itself [67] and with the metallic filler as discussed before [64].

Figure 7 presents the surfaces of PDMS-MPs composites with 50 and 70 wt.% MPs both in 2D and 3D view. Significant differences between the surface before and after incubation process are clearly visible. The peaks shown in Figure 7d in the 3D model are a representation of the surface irregularities. The composites before conditioning have a smoother surface than materials after 12 weeks of conditioning in 0.9 wt.% sodium chloride solution. This leads to the conclusion that the smoothness of surface decreases with the time of incubation. Contact between solution and material affects surface roughness, causing more imperfections. We note that the surface is changing in a non-predictable way. To prevent this surface change, PDMS modifications can be applied [68,69,70]. Increased surface roughness implies a greater area on the material, which can have a positive impact for cell proliferation. Moreover, increased total surface area results in reduced contact angle on the examined surface [71].

### 3.3. Rheological Properties

**Figure 8:**
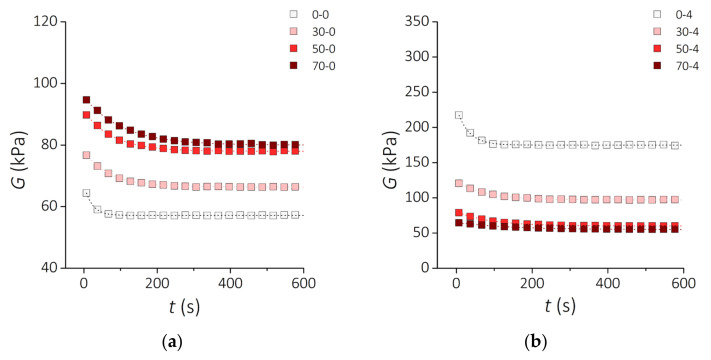
Rheological properties of the PDMS-MPs composites. Relaxation modulus *G* as a function of time for the samples (**a**) before incubation and (**b**) after 4 weeks incubation. Square symbols represent the measurement points. Dashed lines show the fit to the standard linear solid model.

Rheological analyses based on relaxation tests and dynamic oscillation tests reveal how the viscoelastic behavior of the developed PDMS-MPs composites depends on the content of MPs and the incubation time in vitro. Figure 8 shows the time evolution of the relaxation modulus *G* for a time span *t* = 600 s in response to a constant shear strain *γ* = 10%. A characteristic feature of these traces is the non-linear decrease of the *G* value over time, which is abrupt at the beginning and then tends to plateau. In Figure 8a it can be seen that the relaxation module raises as the MPs content is increased for samples before incubation. In contrast, after incubating the samples for 4 weeks, the relaxation modulus decreases as the MPs content in the PDMS matrix increases (Figure 8b).

**Figure 9:**
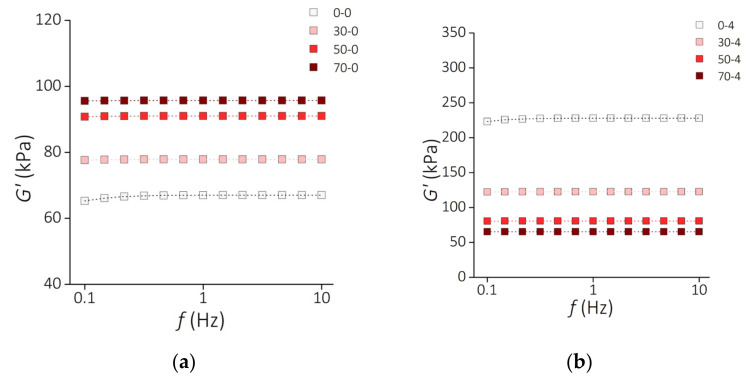
Rheological properties of the PDMS-MP composites. Frequency dependence of the storage modulus *G*′ for the samples (**a**) before incubation and (**b**) after 4 weeks of incubation. Square symbols represent the measurement points. Dashed lines show the fit to the standard linear solid model.

Figure 9 presents the results of the storage modulus *G*′ of the PDMS-MPs composites before incubation (Figure 9a) and samples incubated for 4 weeks (Figure 9b) subjected to oscillating deformations with a constant amplitude of 1% in the frequency range from 0.1 Hz to 10 Hz. The increase in the deformation frequency causes a slight increase in the value of the *G*′ modulus for the PDMS-MPs material. In the low-frequency range (*f* = 0.1–1 Hz) this increase is greater than in the higher range (*f* = 1–10 Hz), where almost constant values of the *G*′ module are observed. Similarly to the relaxation modulus *G*, the storage modulus *G*′ increases with the MPs content for the samples before incubation. This trend is reversed for incubated samples.

**Figure 10:**
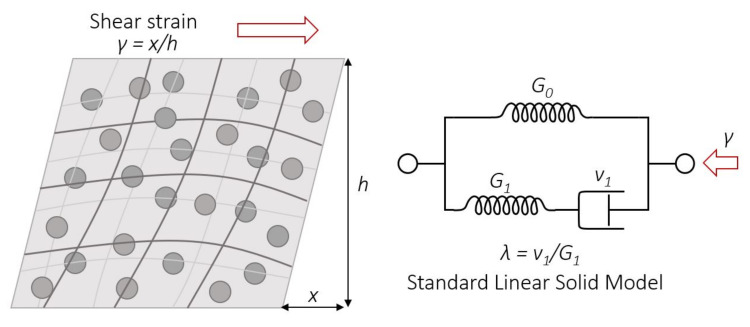
PDMS-MPs composite structure upon deformation (**left**) and scheme of the standard linear solid model (**right**).

To parametrically characterize the obtained results for the relaxation and storage moduli, a standard linear solid model (SLSM) was used. SLSM represents the viscoelastic nature of the material by three components, spring one, spring two, and a dashpot, respectively corresponding to the parameters *G*_0_, *G*_1_, and *v*_1_ (Figure 10).

According to the SLSM, the relaxation module can be written as

(3) $$G\left(t\right)\;=G_{0\;}+G_{1}e^{-t/\lambda }$$

where *G*_0_ and *G*_1_ are the values of the elastic modulus (spring one and spring two), and *λ* is the relaxation time defined as the ratio of the viscosity *v* (dashpot) and *G*_1_ of the SLSM components.

The dependence of the module *G*′ on frequency is described as

(4) $$G^{\prime }\left(f\right)=G_{0}+\frac{G_{1}{\left(fv\right)}^{2}}{1+{\left(fv\right)}^{2}}$$

The calculated SLSM parameters for the obtained results are summarized in Table 6.

As seen in Table 6, the parameters *G*_0_, *G*_1_, and *λ* increase as the content of MPs in the composites before incubation raises, which means that the composites become both stiffer and more viscous. Such enhancement of the mechanical properties with the increase of the filler fraction has been previously described in the literature [4,72]. However, there is no data in the literature on how these properties change with the incubation in biological fluids. Studies of silicone elastomers intended for implants [73] have shown that incubation of these materials in an aqueous environment improves their mechanical properties as a result of the propagation of cross-linking in the polymer. Our study demonstrates that after incubation, the clear PDMS matrix becomes stiffer (*G*_0_ increases from 57,000 Pa to 175,000 Pa) and its relaxation time *λ* increases (*λ* increases from 21.6 s to 31.6 s). A similar behavior is also noticed for composites loaded up to 30 wt.%. In contrast, for composites with 50 and 70 wt.% loading, the *G_0_* value diminishes more than 0 and 30%, respectively. Remarkably, an increase in the relaxation time was observed for all PDMS-MPs composites in this study. These observations point towards structural changes in the material as a result of the interaction with biological fluids. We can hypothesize that several phenomena are taking place. First, swelling of the PDMS matrix without dissolving it, propagation of cross-linking in the polymer and the formation of hydrogen bonds due to water absorption, which increases the stiffness and reduces the relaxation time of the material. Secondly, MP corrosion, loss of cohesive forces with the PDMS matrix, and the formation of micropores around the filler particles occurs, resulting in deterioration of the properties of the PDMS-MPs composites. The latter is caused by pitting corrosion of the filler particles in an aqueous medium containing chloride ions [74].

**Figure 11:**
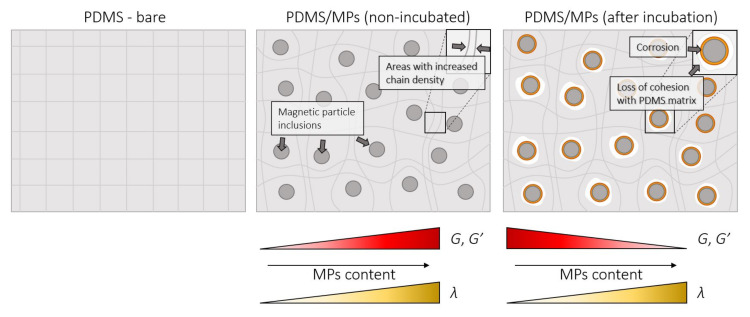
Mechanism of changes in rheological properties in PDMS-MPs composite.

Figure 11 presents the mechanism of changes in rheological properties in PDMS-MPs composites. The supplementation of MPs inclusion to the silicone matrix strengthens the structure of the composite. Additionally, it increases the polymer chain density, which causes the increase of *G* and *G*′ modules and extension of the lambda relaxation time. Incubation in a saline solution environment results in corrosion of the MPs, which results in a loss of cohesion between the MPs and the PDMS matrix. This causes the decrease of values of the *G* and *G*′ modules.

## 4. Summary and Conclusions

We investigated PDMS-MPs composites as well as clear PDMS for their potential use in biomedical applications. We analyzed the influence of the critical variables such as incubation time in a medium solution, temperature, and percentage of the magnetic powder filler. The results obtained for DSC and TGA analysis indicate a lower thermal stability for the composites incubated in 0.9% NaCl solution. It may be considered that the higher percentage of MPs in the PDMS-MPs composite results in a higher temperature to initiate the exothermal reaction. Therefore, the magnetic powder affects the thermal stability of the material. The incubation time reduces the value of the activation energy of the thermal decomposition, as seen for the samples incubated for 12 weeks. In addition, it also decreases the density, smoothness of the surface and the contact angle of the samples. High surface roughness could be beneficial for tissue engineering, but might be counterproductive for drug delivery, as surface modifications might hinder chemical exchanges with the medium. A lower contact angle results in a more hydrophilic surface, which can be of advantage or disadvantage, depending on the application. Materials with a higher percentage of MPs tend to have more stable physicochemical properties. Rheological tests indicate that the silicone matrix becomes stiffer as the incubation time increases. After incubation, the value of the relaxation time increased for all tested materials. The increase of the MPs content in the composite yields decreases relaxation and storage moduli. These results constitute a stepping stone towards studying the viability of these composites as potential smart materials for biomedical applications. The next step will be to further optimize the preparation of materials of this type, magnetize them and test them in a magnetic field.

## Figures and Tables

**Table 1:** Composition of MQFP-14-12 powder.

Element	Concentration Weight (%)	CAS Number
Iron	71.1	7439-89-6
Neodymium	26.0	7440-00-8
Niobium	1.9	7440-03-1
Boron	1.0	7439-89-6

**Table 2:** Designation of examined samples (left column), where the first number indicates the powder content (middle column), and the second number indicates the duration of incubation (right column).

Designation	Percentage of MPs (%)	*t* (weeks)
0-0	0	0
30-0	30	0
50-0	50	0
70-0	70	0
0-1	0	1
30-1	30	1
50-1	50	1
70-1	70	1
0-2	0	2
30-2	30	2
50-2	50	2
70-2	70	2
0-4	0	4
30-4	30	4
50-4	50	4
70-4	70	4
0-8	0	8
30-8	30	8
50-8	50	8
70-8	70	8
0-12	0	12
30-12	30	12
50-12	50	12
70-12	70	12

**Table 3:** Phenomenological data of the thermal decomposition of a clear PDMS and PDMS-MPs-based composites.

Sample	*T*_1 wt.%_ (°C)	*T*_5 wt.%_ (°C)	*T_peak_* (°C)	*Deriv. m* (%/°C)	Residue at 950 °C (wt.%)
0-0	252.71	360.69	534.72	0.6643	40.57
0-1	261.69	377.69	528.16	1.0600	30.83
0-2	263.37	380.21	517.23	0.6242	32.21
0-4	266.73	373.49	700.48	2.7580	25.43
0-8	257.60	373.93	535.97	0.6513	35.2
0-12	258.27	374.60	501.76	1.0943	18.85
30-0	265.89	397.03	468.48	0.2105	60.5
30-1	268.41	404.59	490.33	0.2624	55.86
30-2	275.98	413.84	505.46	0.1956	60.58
30-4	249.92	401.23	479.41	0.2592	54.57
30-8	269.36	404.86	494.95	0.2323	53.15
30-12	184.05	396.79	497.65	0.2477	57.23
50-0	293.00	440.21	476.04	0.1333	74.57
50-1	288.59	437.38	477.72	0.1488	72.02
50-2	277.66	434.15	480.25	0.1454	71.59
50-4	281.02	431.49	483.61	0.1487	66.43
50-8	281.14	429.06	480.84	0.2217	65.82
50-12	224.65	418.30	477.47	0.1777	64.76
70-0	326.42	472.68	485.35	0.1178	85.09
70-1	313.81	461.75	466.80	0.0681	82.82
70-2	298.88	438.48	616.42	0.0991	82.89
70-4	310.44	457.55	465.96	0.0887	82.69
70-8	305.84	479.49	590.44	0.1450	82
70-12	299.96	459.99	469.03	0.0845	82.29

**Table 4:** Maximum temperature (*T_max_*) for each heating rate (*k*) and activation energy (*E_a_*) for 70 wt.% PDMS-MPs composites just after the composite synthesis (70-0) and after 12 weeks of incubation (70-12).

Sample	*k* (°C/min)	*T_max_* (°C)	*T_max_* (K)	*E_a_* (kJ/mol)
70-0	5	460.86	734.01	559.23
	10	466.17	739.32	
	20	471.88	745.03	
70-12	5	440.09	713.24	136.53
	10	464.54	737.69	
	20	481.11	754.26	

**Table 5:** Surface roughness *Sa* of the studied materials upon incubation. The standard deviation σ_Sa_ is listed as well.

Sample	*Sa* (µm)	σ_Sa_ (µm)
0-0	0.103	0.0087
0-12	0.753	0.0237
30-0	0.095	0.0138
30-12	0.099	0.0046
50-0	0.091	0.0016
50-12	0.228	0.0076
70-0	0.128	0.0754
70-12	0.958	0.0271

**Table 6:** SLSM parameters for data from the relaxation modulus and oscillatory tests with fit *R*^2^ > 0.98.

MPs Content in PDMS (%)	*G*_0_ (Pa)		*G*_1_ (Pa)		*λ* (s) = *v*_1_/*G*_1_	
	Non-Incubated	Incubated	Non-Incubated	Incubated	Non-Incubated	Incubated
0	57,000	175,000	10,000	52,900	21.60	31.60
30	66,360	97,000	11,500	25,560	70.00	80.00
50	78,000	60,000	13,000	20,600	78.00	91.00
70	80,000	55,000	15,700	10,115	104.00	138.00
